# Risk Factor Identification for Delayed Gastric Emptying after Distal Pancreatectomy—An Evaluation of 1688 Patients Based on the German StuDoQ|Pancreas Registry

**DOI:** 10.3390/jcm11195539

**Published:** 2022-09-21

**Authors:** Tim Fahlbusch, Philipp Höhn, Carsten Klinger, Jens Werner, Tobias Keck, Helmut Friess, Jörg Köninger, Thomas W. Kraus, Guido Alsfasser, Winfried Padberg, Jörg-Peter Ritz, Waldemar Uhl, Orlin Belyaev

**Affiliations:** 1 Department of General and Visceral Surgery, St. Josef Hospital, Ruhr-University Bochum, 44801 Bochum, Germany; 2 Deutsche Gesellschaft für Allgemein-und Viszeralchirurgie, 10117 Berlin, Germany; 3 Department of General, Visceral, and Transplant Surgery, University Hospital Munich, LMU, 80539 Munich, Germany; 4 Department of Surgery, University Hospital Schleswig-Holstein, Campus Lübeck, 23562 Lübeck, Germany; 5 Klinik und Poliklinik für Chirurgie, Klinikum Rechts der Isar, TUM München, 80333 München, Germany; 6 Klinik für Allgemein-, Viszeral-, Thorax- und Transplantationschirurgie, Katharinenhospital Stuttgart, 70174 Stuttgart, Germany; 7 Allgemein-, Viszeral- und Minimalinvasive Chirurgie, Nordwestkrankenhaus Frankfurt, 60488 Frankfurt am Main, Germany; 8 Klinik und Poliklinik für Allgemein-, Viszeral-, Thorax-, Gefäß- und Transplantationschirurgie, Universitätsmedizin Rostock, 18057 Rostock, Germany; 9 Allgemein-, Viszeral-, Thorax-, Transplantations- und Kinderchirurgie, Universitätsklinikum Gießen Marburg, Standort Gießen, 35392 Gießen, Germany; 10 Klinik für Allgemein- und Viszeralchirurgie, Helios Kliniken Schwerin, 19055 Schwerin, Germany

**Keywords:** pancreatic surgery, postoperative complications, delayed gastric emptying, distal pancreatectomy, StuDoQ, morbidity, postoperative pancreatic fistula

## Abstract

Delayed gastric emptying (DGE) ranks as one of the most frequent complications in pancreatic surgery. It leads to increased costs for healthcare systems, lengthened hospital stays and reduced quality of life. Data about DGE after distal pancreatectomy (DP) are scarce. The StuDoQ|Pancreas registry of the German Society of General and Visceral Surgery provided data of patients who underwent distal pancreatectomy from 1 January 2014 to 31 December 2018. The retrospective evaluation included comprehensive data: 1688 patients were enrolled; DGE occurred 160 times (9.5%); grade “A” was reported for 98 (61.3%), grade “B” for 41 (25.6%) and grade “C” for 21 (13.1%) patients. In univariate analysis pancreatic fistulas were associated with higher frequencies of intraabdominal abscesses (9.1% vs. 2%, *p* > 0.001), postpancreatectomy haemorrhage (8.1% vs. 3.7%, >0.001) and DGE (14.5% vs. 6%, *p* < 0.001). According to multivariate analysis, “abscesses with invasive therapy” (*p* < 0.001), “other surgical complications” (*p* < 0.001), prolonged “stays in ICU” (*p* < 0.001), lengthened duration of surgery (*p* < 0.001) and conventional surgery (*p* = 0.007) were identified as independent risk factors for DGE. Perioperative and postoperative factors were identified as risk factors for DGE. Following research should examine this highly relevant topic in a prospective, register-based manner. As there is no causal therapy for DGE, its avoidance is of major importance.

## 1. Introduction

During the last decades, mortality in pancreatic surgery could be highly decreased. Experienced surgical centres report mortality rates of 0–6% [1]. Nevertheless, perioperative morbidity remains at a high level [2].

The most frequent complication is delayed gastric emptying (DGE), which occurs in up to 80% of cases after pancreatic head resections [3]. A lengthened hospital stay, high costs for the healthcare system, a reduced quality of life and a potential delay of an adjuvant cancer therapy may be caused by DGE [4].

According to the International Study Group for Pancreatic Surgery (ISGPF), DGE is defined by the amount of days for which a nasogastric tube is required and no solid food can be digested [5].

DGE following pancreaticoduodenectomy (PD) has been extensively examined. Hormonal dysbalances due to the resection of the duodenum, gastric denervation and mechanical alterations are suspected causes for the occurrence of DGE [6]. Various surgical techniques, including Billroth II/Roux-en-Y and antecolic/retrocolic reconstructions, have been analyzed. Reported results differed widely [1,5,7].

The German Society of General and Visceral Surgery (DGAV) initiated a national registry (Studien-, Dokumentations- und Qualitätszentrum, StuDoQ) for pancreatic surgery in 2013 (StuDoQ|Pancreas). It provides extensive information from German and foreign pancreatic surgery centers [8]. Data about indications, demographics and perioperative outcomes after various surgical procedures have been collected.

A previous StuDoQ|Pancreas-based analysis of DGE following pancreaticoduodenectomy (PD) identified higher age, longer duration of surgery, reconstruction as pancreaticogastrostomy (PG), postoperative pancreatic fistula (POPF), insufficiency of the hepaticojejunostomy and other surgical complications as risk factors for the occurrence of DGE [2]. Additionally surgical techniques, sepsis or intraabdominal abscesses are discussed as risk factors for DGE in the literature [7,9,10]. As there is no known causal therapy, the prevention of DGE should be beneficial.

In literature, the frequency of DGE after DP is low compared to DGE after PD. According to the living meta-analysis of the evidence map on pancreatic surgery of the ISGPS, incidence levels of 6% vs. 15% are stated [11]. In single center studies, rates of 25% are reported. Its severity is less distinct [12]. Yet, data are scarce and studies rarely reported. Therefore, we aim to increase knowledge about this clinically highly relevant issue by this register-based, retrospective analysis of a large quantity of patients on behalf of the nationwide registry.

## 2. Materials and Methods

### 2.1. The StuDoQ|Pancreas Registry

The StuDoQ|Pancreas registry is a German nationwide database, which was originated by the “Deutsche Gesellschaft für Allgemein- und Viszeralchirurgie” (DGAV, German Society of General and Visceral Surgery). Its aim is the assessment and monitoring of the quality of pancreatic surgery in Germany. After written consent of all patients included in the study, information from over 50 high-volume pancreatic surgery centers were gathered in an online tool. All data were pseudonymized and analyzed in a retrospective manner. All StuDoQ|Pancreas information was certified each year and cross-checked regularly. All cases of DP performed from 1 January 2014 to 31 December 2018were enlisted, including demographics, surgical techniques, histopathological and perioperative data. Patients with an unknown DGE status or who underwent a surgical procedure other than DP were not taken into account.

### 2.2. Definitions

DP were enlisted as Warshaw and Strasberg procedures. Lymphadenectomy (LAD), DGE, postpancreatectomy hemorrhage (PPH) and chyle leakage were analyzed according to the grading system of the International Study Group for Pancreatic Surgery (ISGPS) [5,13,14,15]. Complications and morbidity were assessed using the Clavien–Dindo Classification [16]. The term “abscesses with invasive therapy” referred to all therapeutical options other than drugs.

### 2.3. Statistical Analysis

Statistics were calculated using SPSS V21.0 (IBM Corp. Released 2015, IBMStatistics for Windows, Version 23.0., IBM Corp., Armonk, NY, USA) and WinPepi (Pepi-for-Windows) [17]. A two-sided significance level of 0.05 was applied. Scale variables were assessed by mean and range; categorical variables by absolute count and percentages. Student’s *t*-test, Mann–Whitney-U, Kruskal–Wallis and chi^2^ test were performed for univariate analyses. A multivariate logistic regression model was calculated for statistically significant associations with DGE.

### 2.4. Ethical Statement

The study was performed in accordance with the Declaration of Helsinki (as revised in 2013) and approved by the ethics committee of the Ruhr-University Bochum, Germany (Reg. Nr. 20-7140-bio).

## 3. Results

A total of 1688 patients were enrolled in the study, all of which underwent a distal pancreatectomy and were monitored by the StuDoQ|Pancreas registry. A total of 803 (47.6%) patients were male and 885 (52.4%) were female. The mean age in the entire study population was 63.1 ± 13.8. The average BMI was 25.9 ± 5 kg/m^2^. The most common preoperative clinical symptoms were pain (38.6%, n = 651), nausea (9.7%, n = 164) and hypoglycaemia (1.8%, n = 30). A total of 43.4% of patients (n = 734) were classified as ASA (American Society of Anaesthesiologists) III or higher, whereas 7.8% (n = 132) of patients were assigned to ASA I and 48.8% of cases (n = 823) to ASA II. The mean postoperative hospitalization amounted to 17.3 ± 13.4 days and the mean length of stay in the ICU was 2.75 ± 6.8 days. The average duration of surgery was 217.3 ± 83.9 min. In 69.7% (n = 1175) of cases, a primarily open approach for the DP was chosen, followed by laparoscopy (18.1%, n = 305), secondarily open procedures (6.8%, n = 114) and laparoscopic assistance (5.5%, n = 93). Table 1 highlights surgical data.

A laparoscopic approach was rather chosen for benign diseases (24.1% vs. 7.9%, *p* < 0.001) and patients with lower ASA scores (ASA ≤ 2, 20.5% vs. 14.9%, *p* = 0.001). Patients in the “open surgery” group were older (64.4 vs. 60.1 years, *p* = 0.001) and slightly lighter (BMI 25.7 vs. 26.1, *p* = 1). The laparoscopic approach was associated with an earlier discharge (13.6 ± 10.9 vs. 18.7 ± 14.2 days, *p* < 0.001), a shorter duration of surgery (200.9 ± 71.5 vs. 217.8 ± 87.8 min, *p* = 0.024) and a shorter stay in the ICU (1.5 ± 3.1 vs. 3.2 ± 6.8 days, *p* < 0.001). The following Table 2 presents patients’ data and outcomes according to the technique.

An overall 30-day mortality of 1.1% (n = 19) was reported. The majority of the patients died of non-surgical reasons (n = 10, 52.6%).

A pancreatic ductal adenocarcinoma was the most common tumor (37.3%, n = 626) the DPs were performed for. In total, 642 patients (38.3%) were operated on within the context of malignant entities. Rare tumorous diagnoses were cystadenocarcinomas (0.8%, n = 13) and intrapapillary mucinous neoplasm-associated carcinomas (0.2%, n = 3). The majority of lesions in the study population were benign (61.7%, n = 1032). The most frequent diagnoses were pancreatic neuroendocrine neoplasms (18.7%, n = 315), followed by chronic pancreatitis (11.5%, n = 193) and intrapapillary mucinous neoplasms (12.4%, n = 208). Mucinous cystic neoplasms (6%, n = 101), serous cystic neoplasms (5.7%, n = 95) and pseudocysts (2.7%, n = 45) were more rarely reported.

Conventional surgery was more often chosen for malignant entities, whereas laparoscopic approaches were more common for surgery on benign tumors and chronic pancreatitis. An overview is to be found in the following Table 3.

Standard lymph node dissections were reported in 90.9% (n = 1185), and 72.1% (n = 782) of pancreatic tissues were described as “soft”. Blind closures of the pancreatic remnant were performed in 87.7% of cases (n = 1368). This strategy was pursued in 945 cases in the “open surgery” group (86.6%) and in 247 cases in the “laparoscopic surgery” group (89.2%). Pancreaticogastrostomies were only reported for open procedures (2.5%, n = 29). POPF occurred in 40.3% (n = 681) of patients. A blind closure led to the highest share of POPF (40.9, n = 559). PG (32.3%, n = 10) and PJ (36%, n = 58) were associated with lower levels of POPF. PPHs were found in 5.4% of patients (n = 92), and abscesses needed to be treated invasively in 4.9% (n = 82) of cases. DGE occurred 160 times (9.5%), mainly as grade “A” (61.3%, n = 98). Grade “B” was reported for 41 (25.6%) and grade “C” for 21 (13.1%) patients.

A comparison of patients suffering from DGE and free of DGE is to be found in Table 4.

Intraabdominal abscesses occurred more often in patients with POPF than in patients without POPF (9.1% vs. 2%, *p* > 0.001). POPF were also linked with higher frequencies of PPH (8.1% vs. 3.7%, >0.001) and DGE occurred more often in patients with POPF (14.5% vs. 6%, *p* < 0.001).

The multivariate logistic regression analysis a longer duration of surgery, a prolonged stay in ICU, open surgery, a postoperative “abscess with invasive therapy” and postoperative “other surgical complications” identified independent risk factors for DGE (see Table 5).

## 4. Discussion

DGE is the most common complication in pancreatic surgery. After PD, it is reported to occur in up to 80% of cases [3]. It leads to higher costs for healthcare systems due to a prolonged hospital stay and a reduced quality of life [4]. Furthermore, an early initiation of an adjuvant chemotherapy might be at risk.

In literature, DGE after PD has been widely analyzed. Contrastingly, DGE after DP has been seldomly examined. According to the available studies, the rates of DGE are lower following DP. Glowka et al. reported a frequency of 24% and a majority of mild grades [7]. In our study, we report an occurrence of DGE in 9.5% of cases. Grade A was the most common appearance (61.3%). DGE patients were older and more often female in the compared group, which was also reported in the literature [7].

The background of DGE is still a subject of discussion. Hormonal dysbalances after the resection of the duodenum, gastric ischemia and denervation due to the mobilization and lymphadenectomy are proposed causes for the occurrence of DGE after PD [18]. Furthermore, associations with reconstructional aspects (Billroth II vs. Roux en Y, antecolic vs. retrocolic) have been discussed [9,19,20]. These classical risk factors do not apply for DP procedures as the duodenum remains in situ. In the case of a blind closure after DP there is no ante- or retrocolic reconstructional route. A blind closure was by far the most common strategy for the pancreatic remnant (87.7%). Additionally, the stomach is mobilized less in the DP procedure.

Intraabdominal fluids are suspected to increase the risk for DGE [21]. In our study, we found a high frequency of DGE in patients who also suffered from POPF. Especially, intrabdominal abscesses were highly associated with POPF. After multivariate analysis, POPF is in contrast to an abscess no independent risk factor for DGE. Nevertheless, as the abscess might be traced to POPF, the findings in literature can be supported on this matter. The management of the pancreatic remnant is still a subject of discussion. Among other technical approaches, PG and PJ are supposed to reduce to occurrence of POPF, but results in the literature differ [22,23,24]. We found the lowest share of POPF after PG. Due to the rareness of PGs in our study population, the statistical significance might be impaired.

After multivariate analysis, we identified “other surgical complications” as an independent risk factor for DGE. This could be a chyle leakage, gastrointestinal bleeding or pancreatitis in the remnant. Unfortunately, this variable is not specified in StuDoQ|Pancreas and gives room for further, more precise elucidation. “Other surgical complications” also was found to be a risk factor for DGE after DP [2].

Histological results were not associated with the frequency of DGE, supporting findings in the literature [6].

DGE after DP is, in contrast to PD, rarely examined in literature. To our knowledge, this study accesses the data of the largest number of patients so far. Furthermore, some studies do not refer to the ISGPS definitions (due to the time of publication) leading to impaired comparability [25,26]. Other studies present their results only related to major vascular resections [27].

Still, the significance of our work is reduced by its design. All information was gathered prospectively, but the analysis was retrospective. Furthermore, there are data which StuDoQ|Pancreas is not able to provide. The technique of blind closure or the use of drainages has not been specified. The participating centers report grades of DGE. A more precise evaluation could be enabled by the provision of the exact postoperative day a nasogastric tube is removed or inserted.

Nevertheless, the StuDoQ|Pancreas registry offers the unique possibility to evaluate the largest quantity of patients’ data in this context so far. It is an important tool to elucidate the clinically highly important issue in pancreatic surgery. In absence of a causal therapy, it emphasizes the importance of the prevention of DGE.

## Figures and Tables

**Table 1:** Detailed demonstration of surgical data; abbreviations: ICU, intensive care unit; A, B, C, grades of severity of postoperative pancreatic fistulas, delayed gastric emptying and postpancreatectomy hemorrhage.

Characteristics (n = 1688)	Value
Approach Laparoscopic Laparoscopically assisted Primarily open Secondarily open	18.1% (n = 305) 5.5% (n = 93) 69.7% (n = 1175) 6.8% (n = 114)
Duration of surgery (minutes)	217.3 ± 83.9
ICU stay (days)	2.75 ± 6.8
30-day survival	98.9% (n = 1669)
Lymph node dissection Standard Extended None	76.2% (n = 1185) 7.5% (n = 118) 16.2% (n = 252)
Pancreatic consistency Soft Hard	72.1% (n = 782) 27.9% (n = 302)
Pancreatic closure Blind Pancreaticogastrostomy Pancreaticojejunostomy	87.7% (n = 1368) 2% (n = 31) 10.3% (n = 161)
Postoperative pancreatic fistula Biochemical leakage B C None	15.5% (n = 261) 18.5% (n = 313) 6.3% (n = 107) 59.7% (n = 1007)
Delayed gastric emptying A B C None	5.8% (n = 98) 2.4% (n = 41) 1.2% (n = 21) 90.5% (n = 1528)
Postpancreatectomy hemorrhage A B C None	1.2% (n = 20) 2.4% (n = 41) 1.8% (n = 31) 94.5% (n = 1596)
Abscess with invasive treatment	4.9% (n = 82)

**Table 2:** Patients’ and surgical data apportioned by surgical technique; abbreviations: lap. Assisted, laparoscopically assisted; prim. Open, primarily open; sec. open, secondarily open; BMI, body mass index; ICU, intensive care unit; ASA, American Society of Anaesthesiologists; ben., benigne; mal., malignant; POPF, postoperative pancreatic fistula; DGE, delayed gastric emptying.

Value	Laparoscopic (n = 305)	Lap. Assisted (n = 93)	Prim. Open (n = 1175)	Sec. Open (n = 114)	*p*-Value
Age	60.14 ± 15.6	58.7 ± 15	64.38 ± 13	61.5 ± 13.4	<0.001
BMI [kg/m^2^]	26.2 ± 5.5	26.19 ± 5.1	25.7 ± 4.7	27.2 ± 5.1	0.019
Duration of surgery	200.9 ± 71.5	230 ± 68.7	217 ± 87.8	245.3 ± 74.5	<0.001
Stay in ICU	1.5 ± 3.1	2.2 ± 4.3	3.2 ± 6.8	2.0 ± 4.2	<0.001
ASA ≤2 ≥3	64.3% (n = 196) 35.7% (n = 109)	66.7% (n = 62) 33.3% (n = 31)	54% (n = 634) 46% (n = 541)	54.4% (n = 62) 45.6% (n = 52)	0.001
Tumor ben. mal.	83.3% (n = 254) 16.7% (n = 51)	88.2% (n = 82) 11.8% (n = 11)	53.5% (n = 629) 46.5% (n = 546)	68.4% (n = 78) 31.6% (n = 36)	<0.001
POPF Biochemical leak B C None	23.9% (n = 73) 13.4% (n = 41) 7.5% (n = 23) 55.1% (n = 168)	26.9% (n = 25) 18.3% (n = 17) 2.2% (n = 2) 52.7% (n = 49)	12.6% (n = 148) 19.4% (n = 228) 6.5% (n = 76) 61.5% (n = 723)	13.2% (n = 15) 22.8% (n = 26) 5.3% (n = 6) 58.8% (n = 67)	0.042
DGE A B C none	3.9% (n = 12) - 0.3% (n = 1) 95.7% (n = 292)	5.4% (n = 5) 1.1% (n = 1) - 93.5% (n = 87)	6.2% (n = 73) 3.1% (n = 36) 1.5% (n = 18) 89.2% (n = 1048)	7.0% (n = 8) 2.6% (n = 3) 1.8% (n = 2) 88.6% (n = 101)	<0.001

**Table 3:** Histological diagnosis apportioned by the surgical technique; PDAC, pancreatic ductal adenocarcinoma; IPMN, intrapapillary mucinous neoplasms; MCN, mucinous cystic neoplasms; SCN, serous cystic neoplasms; pNEN, pancreatic neuroendocrine neoplasms; CP, chronic pancreatitis.

Diagnosis	Laparoscopic n = 305	Lap. Assisted n = 93	Prim. Open n = 1175	Sec. Open n = 114	*p*-Value
PDAC	14.9% (n = 45)	11.8% (n = 11)	45.6% (n = 533)	32.1% (n = 36)	<0.001
IPMN	18.9% (n = 57)	21.5% (n = 20)	9.4% (n = 110)	18.8% (n = 21)	<0.001
MCN	12.3% (n = 37)	7.5% (n = 7)	3.9% (n = 46)	9.8% (n = 11)	<0.001
SCN	8.9% (n = 27)	12.9% (n = 12)	4.4% (n = 52)	3.6% (n = 4)	0.004
pNEN	6.3% (n = 19)	34.4% (n = 32)	16.3% (n = 191)	14.3% (n = 16)	<0.001
CP	7% (n = 21)	6.5% (n = 6)	12.7% (n = 149)	15.2% (n = 17)	0.005

**Table 4:** Univariate evaluation of the DGE and non-DGE group; significant *p*-values are printed in bold letters; abbreviations: ICU, intensive care unit; PPH, postpancreatectomy hemorrhage; POPF, postoperative pancreatic fistula; DGE, delayed gastric emptying.

Characteristics	DGE Positive (n = 160)	DGE Negative (n = 1528)	*p*-Value
Age (years)	65.29 ± 13.3	62.88 ± 13.8	0.029
Sex Male Female	74 (46.3%) 86 (53.8%)	729 (47.7%) 799 (52.3%)	0.725
Duration of surgery	254.1 ± 105.7	213.4 ± 80.4	<0.001
Duration of stay in ICU	6.14 ± 9.7	2.4 ± 5.5	<0.001
PPH A B C None	3 (1.8%) 9 (5.6%) 8 (5%) 140 (87.5%)	17 (1.1%) 32 (2.1%) 23 (1.5%) 1456 (95.3%)	<0.001
Approach Laparoscopic Laparascopically assisted Open Secondarily open	13 (8.1%) 6 (3.8%) 127 (79.4%) 13 (8.1%)	292 (19.1%) 87 (5.7%) 1048 (68.6%) 101 (6.6%)	0.017
Postoperative abscess/invasive therapy	29 (18.1%)	53 (3.5%)	<0.001
Wound site infection	22 (13.8%)	98 (6.4%)	0.42
POPF Biochemical leak B C none	23 (14.4%) 42 (26.3%) 30 (18.8%) 65 (40.5%)	238 (15.5%) 271 (17.7%) 77 (5%) 942 (61.3%)	<0.001
Other surgical complications	32 (20%)	107 (7%)	0.015

**Table 5:** Results of the multivariate analysis; ICU, intensive care unit.

Characteristics	*p*-Value
Duration of surgery	<0.001
Stay in ICU	<0.001
Open surgery	0.007
Abscess with invasive therapy	<0.001
Other surgical complications	<0.001

## Data Availability

All data are property of the German Society of General and Visceral Surgery, but can be consulted upon request.
